# In Vitro Digestion and Gut Microbiota Fermentation of the Anticancer Marine Drug BG136: Stability and Biotransformation Investigation

**DOI:** 10.3390/md23040156

**Published:** 2025-04-03

**Authors:** Xintong Li, Shuying Xu, Baiyuan Chen, Pengcheng Gao, Youjing Lv, Qingsen Shang, Guangli Yu, Guoyun Li

**Affiliations:** 1 Key Laboratory of Marine Drugs, Ministry of Education, Shandong Key Laboratory of Glycoscience and Glycotherapeutics, School of Medicine and Pharmacy, Ocean University of China, Qingdao 266003, China; lixintong20000723@163.com (X.L.); 17syxu@alumni.stu.edu.cn (S.X.); cbyngu@163.com (B.C.); gaopengchengsir@163.com (P.G.); 2015315@ouc.edu.cn (Y.L.); shangqingsen@ouc.edu.cn (Q.S.); 2 Laboratory for Marine Drugs and Bioproducts, Qingdao Marine Science and Technology Center, Qingdao 266237, China

**Keywords:** BG136, digestion, fermentation, gut microbiota, SCFAs

## Abstract

BG136, a β-1,3/1,6-glucan derived from *Durvillaea antarctica*, is an injectable anticancer drug and has entered Phase II clinical trials. Rational oral formulation design is a pivotal focus for our future drug development research; therefore, elucidating the gastrointestinal fate of BG136 becomes imperative. This study investigated the stability and biotransformation of BG136 via in vitro digestion and gut microbiota fermentation. The results confirmed BG136’s structural integrity, resistance to degradation in a highly acid environment and by gastrointestinal tract enzymes. In contrast, BG136 was degraded by intestinal bacteria into mid-size fragments along with smaller oligosaccharides. Additionally, the biotransformation process notably elevated total short-chain fatty acids (SCFAs) to 38.37 ± 3.29 mM, representing a 59.4% increase versus controls (24.08 ± 2.29 mM), with propionic acid exhibiting the most substantial increase. Meanwhile, the process was accompanied by significant microbial regulation, including an increase in beneficial genera (*Lactobacillus*, *Enterococcus*) and a reduction in *Lachnoclostridium* populations. Overall, these findings systematically map the oral bioavailability challenges and prebiotic potential of BG136, highlighting its microbiota-modulating capacity through species-specific ecological regulation, providing insights into oral drug development for BG136.

## 1. Introduction

The marine ecosystem represents a rich reservoir of biologically active compounds, among which polysaccharides have versatile applications, notably in the fields of biomedicine, food science, and materials engineering [1,2]. BG136, a β-1,3/1,6-glucan from *Durvillaea antarctica*, has a homogeneous molecular weight of 3–6 kDa [3]. Previous studies have shown that BG136 has promising potential in tumor growth inhibition and anti-tumor metastasis [4,5]. Recently, BG136 for intravenous injection has entered clinical trials in China (clinical approval no.: 2022LP02021) as the first marine polysaccharide drug targeting immuno-oncology and anti-tumor therapy.

However, compared to intravenous administration, oral administration is the most common drug delivery method in pharmacotherapy, offering advantages such as convenience, safety, low cost, and high patient compliance [6]. Furthermore, in recent years, great progress has been made in the development of oral drugs with β-glucan as the active ingredient [7]. Zhang et al. showed that *Laminaria japonica* polysaccharide exerted a lipid-lowering effect in high-fat diet (HFD) mice via modulating hepatic gene expressions of AMPK and HMGCR [8]. Zou et al. found that β-glucan extracted from autumn-planting mushroom was able to inhibit tumor growth and target tumor sites by oral administration [9]. In addition, previous studies have shown that oral β-glucan can also treat inflammatory bowel disease [10,11]. And studies in our laboratory have also shown that oral administration of BG136 can effectively inhibit the growth of S-180 tumor cells. Accordingly, the success of injectable BG136 warrants the development of alternative administration routes to enhance patient convenience and compliance.

With the advancement in research on the oral pharmacological activity of polysaccharide drugs, there have been numerous reports on the oral absorption pathways of polysaccharide prototypes in the intestinal epithelial cells, such as *Lycium barbarum* polysaccharides [12], *Pseudostellaria heterophylla* polysaccharides [13], and *Ganoderma lucidum* polysaccharides [14]. Nevertheless, critical factors such as gastrointestinal digestion and microbial utilization substantially constrain the comprehensive understanding of the oral absorption forms for polysaccharides. And it is worth noting that the pharmacological activity of polysaccharides is intrinsically linked to their metabolic processes within the gastrointestinal tract [15].

Polysaccharides are classified as digestible and indigestible polysaccharides [15]. Digestible polysaccharides, such as mulberry fruit polysaccharides [16] and *Holothuria leucospilota* polysaccharides [17], are degraded in the gastrointestinal tract and absorbed into the blood circulation. In contrast, indigestible polysaccharides (e.g., konjac glucomannan [18]) are transported to the colon, which harbors a large and complex microbial community known as the gut flora [19,20]. Notably, these gut microbiota encode numerous carbohydrate-active enzymes (CAZymes), including glycoside hydrolases (GHs), polysaccharide lyases (PLs), and carbohydrate esterases (CEs), which collectively assemble into polysaccharide utilization loci (PULs). These specialized sites facilitate the translocation and subsequent catabolism of non-digestible polysaccharides by the intestinal microbial community [21]. Our previous studies elucidated that *Bacteroides uniforms L8* secretes β-agarase and another glycoside hydrolase to degrade agarose into D-galactose [22], while in *B. ovatus E3,* we identified PL8, PL29, PL35, PL33, and GH88 as active enzymes related to the degradation of hyaluronic acid [23].

Indigestible polysaccharides, while undergoing microbial metabolism, possess the dual capacity to regulate the gut microbial composition as well as produce beneficial metabolites, especially SCFAs. Synthesized by gut microbiota, SCFAs are monocarboxylic acids containing fewer than six carbon atoms [24]. Substantial evidence has shown that acetic acid energizes intestinal epithelial cells, regulates immune cell activity, and maintains the integrity of the intestinal barrier [25]. Additionally, propionic acid improves fatty acids in the liver and increases insulin sensitivity [26]. Our prior investigations demonstrated that polysaccharide from *E. clathrata* produced more acetate acid and promoted the growth of bacteria with documented anti-colitis properties, such as *B. thetaiotaomicron*, *B. ovatus*, and *B. uniformis* [27]. Moreover, we also confirmed that fucoidan exhibited structure-dependent modulation of gut microbiota composition, characterized by increased *Lactobacillus* and *Ruminococcaceae* abundance with concomitant reduction in *Peptococcus* [28].

Overall, gastrointestinal drug metabolism, including microbial and host-mediated processes, plays a critical role in the development of oral therapeutics. However, conducting in vivo metabolism studies of orally administered drugs remains challenging, primarily due to the difficulties in obtaining representative gastrointestinal fluid samples, detecting trace-level metabolites, and controlling biotransformation timelines. In contrast, in vitro digestion and fermentation models accurately simulate both drug stability in the gastrointestinal lumen and the metabolic capacity of gut microbiota. These models have gained widespread adoption due to their superior reproducibility and experimental efficiency compared to in vivo approaches [29,30]. Building upon the in vitro simulated digestion and fermentation models, we systematically elucidated the digestion characteristics of BG136 in simulated gastric fluid and small intestinal fluid, explored the biotransformation patterns mediated by gut microbiota, and revealed the intestinal benefits of BG136, aiming to establish a metabolic characterization foundation for developing targeted oral delivery strategies.

## 2. Results

### 2.1. Structural Stability of BG136 During In Vitro Digestion

**Figure 1:**
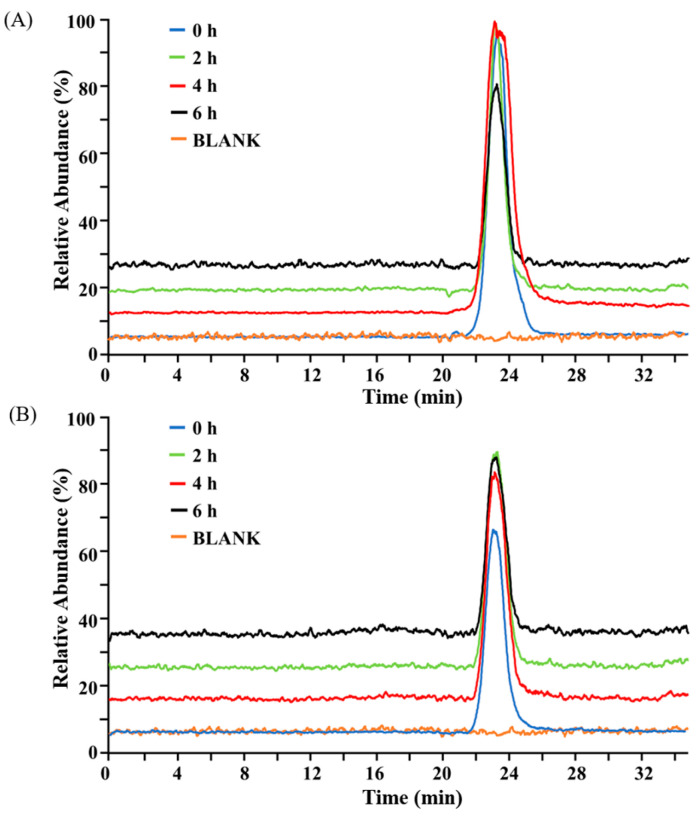
The total ion chromatogram (TIC) of BG136 after digestion in (**A**) simulated gastric fluid and (**B**) simulated small intestinal fluid for 0, 2, 4, and 6 h, respectively.

The digestive characteristics of oral pharmaceuticals in gastrointestinal fluids represent a prominent consideration in formulation development, underscoring the necessity of characterizing drug metabolism via in vitro gastrointestinal digestion models. To assess whether BG136 undergoes degradation in the stomach and small intestine, we analyzed its molecular weight changes during simulated digestion using high performance size exclusion chromatography-mass spectrometry (HPSEC-MS). HPSEC enables molecular weight-dependent separation, thus providing a validated approach to monitor BG136’s structural integrity during digestive processes [31]. As shown in Figure 1, there were no significant alterations in chromatographic behavior observed in either gastric or small intestinal fluids, indicating that BG136 remains stable in gastrointestinal fluids. The observation likely originates from endogenous β-glycosidase deficiency in digestive secretions, rendering β-glycosidic bonds uncleavable [32].

### 2.2. Saccharides Dynamics During In Vitro Fermentation

**Figure 2:**
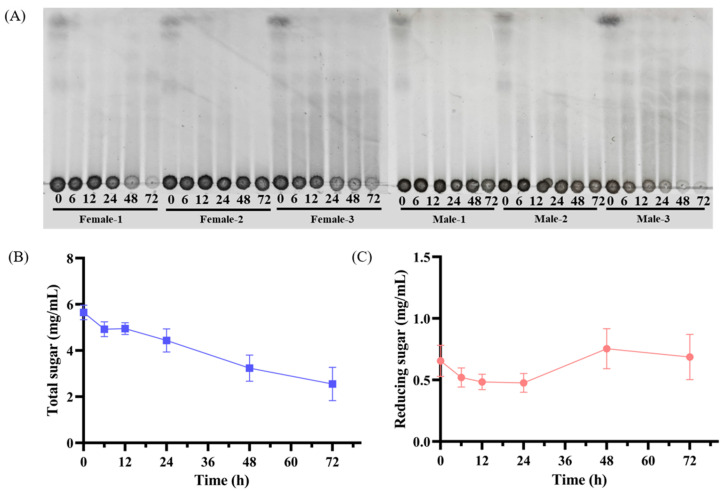
Changes in saccharides during fermentation. (**A**) The thin-layer chromatogram of BG136 in vitro fermentation for 0, 6, 12, 24, 48, and 72 h. Changes in (**B**) total sugar and (**C**) reducing sugar contents during fermentation (*n* = 6).

Following oral administration, polysaccharide metabolism encompasses both digestive processes in gastrointestinal fluids and catabolism mediated by gut microbiota. There is a growing trend to study the fermentability of polysaccharides by gut microbiota due to their indigestible properties [33]. Thin-layer chromatography (TLC) was used to determine whether BG136 would be degraded by the gut flora. It is well known that less polar components exhibit greater mobility across the silica plate, while polysaccharides typically remain at the origin due to their high molecular weight and polarity. As shown in Figure 2A, the progressive attenuation of staining intensity at the origin correlated with fermentation duration, providing visual evidence of BG136 degradation and subsequent oligosaccharide generation, which migrated above the origin. Furthermore, it is worth mentioning that significant individual differences exist in the rate and extent of fermentation. It has also been shown that individualized responses of the gut microbiota to indigestible polysaccharides contribute to variability in health outcomes [34].

Polysaccharide depolymerization is characteristically associated with elevated reducing sugar levels [35]. The changes in total sugar content and reducing sugar content reflect the consumption and utilization of carbohydrates by the intestinal microbiota [36]. As depicted in Figure 2B, the total sugar content decreased gradually during fermentation, from 5.65 ± 0.32 mg/mL (0 h) to 2.55 ± 0.72 mg/mL (72 h), indicating that the intestinal microbiota consistently utilized BG136. The reducing sugar content increased from 0.48 ± 0.08 mg/mL to 0.76 ± 0.16 mg/mL between 24 and 48 h, but at the late stage of fermentation, it decreased to 0.69 ± 0.18 mg/mL at 72 h (Figure 2C), potentially due to a decrease in intestinal flora vitality and a higher utilization of oligosaccharides compared to the degradation of polysaccharides [37]. These results suggest that the degradation process of BG136 and utilization of degraded oligosaccharides by the intestinal flora occur in parallel. The fermentability data were calculated by Formula (1) and varied from 56.77% to 86.75%, with a mean ± SD of (65.66 ± 13.87)%. The pronounced interindividual variations could be attributed to host-specific metabolic signatures of gut microbiota, resulting in differential efficiency in substrate utilization [38]. Furthermore, essential physiological factors, such as the luminal pH and gastrointestinal transit time, can significantly influence the fermentation dynamics by modulating the microbiome’s responsiveness to pharmaceutical agents [39].

### 2.3. Structural Modifications of BG136 During In Vitro Fermentation

**Figure 3:**
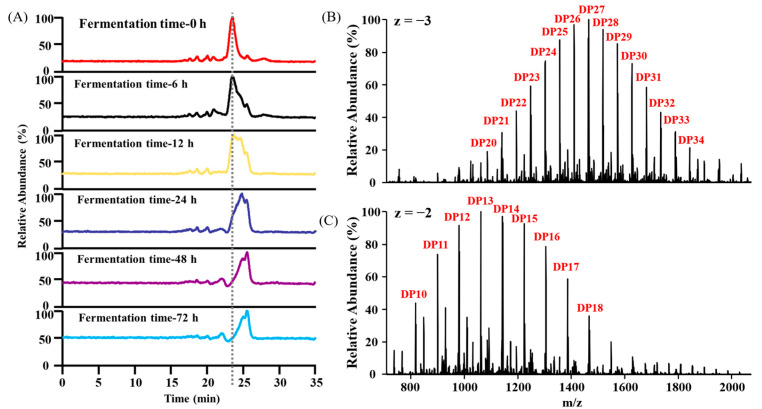
HPSEC-MS detection of fecal fermentation samples. (**A**) The TIC of BG136 fermented for 0, 6, 12, 24, 48, and 72 h, respectively. Backward shift of peak was indicative of hydrodynamic volume reduction. (**B**) Primary mass spectra of the chromatographic peak at 0 h [*M* − 3*H*]^3−^. (**C**) Primary mass spectra of the chromatographic peak at 72 h [*M* − 2*H*]^2−^. Decrease in mass-to-charge (*m*/*z*) ratios and charge state (z) transition from −3 to −2 represented molecular weight diminution. DP: polymerization degrees.

While TLC effectively identifies enzymatically hydrolyzed oligosaccharides, its analytical resolution precludes detection of BG136-mediated high-molecular-weight polysaccharide cleavage fragments. To more clearly and intuitively indicate the degree of degradation of BG136 during different fermentation periods, HPSEC-MS was used in this study. As demonstrated in Figure 3A, the TIC profile of BG136 exhibited a pronounced backward shift with prolonged fermentation time. This temporal delay in retention time observed in size exclusion chromatography correlated with molecular weight reduction. Furthermore, comparative mass spectral profiling of BG136 at fermentation onset (0 h) and endpoint (72 h) (Figure 3B,C) demonstrated a significant decrease in the DP of BG136, conclusively demonstrating gut microbiota-mediated depolymerization of BG136.

### 2.4. BG136 Fermentation Promoted the Production of SCFAs

**Figure 4:**
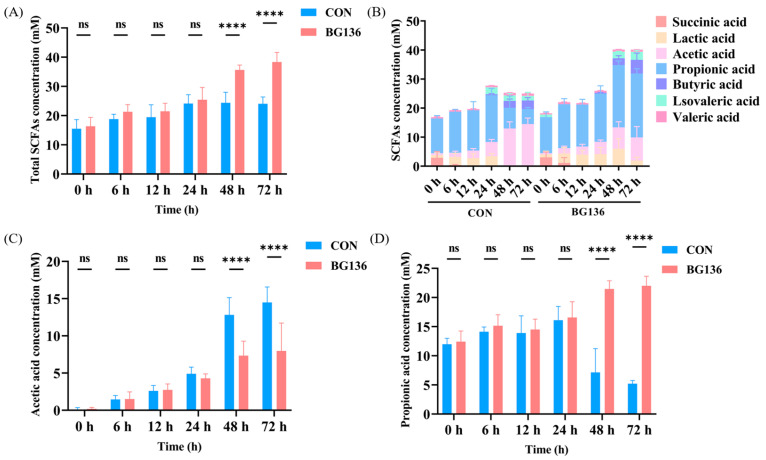
Assessment of the impact of BG136 on the generation of SCFAs. (**A**) Changes in total SCFAs concentration during fermentation. (**B**) The production levels of SCFAs in different time duration, especially (**C**) acetic acid and (**D**) propionic acid (*n* = 6). ns: *p* > 0.05, ****: *p* < 0.0001.

Beyond generating oligosaccharides, polysaccharides fermentation yields a diverse array of metabolites, among which SCFAs serve as crucial energy substrates and signaling molecules [40,41]. As demonstrated in Figure 4A, the total SCFAs concentration in the BG136 group increased from 16.36 ± 3.06 mM (0 h) to 38.37 ± 3.29 mM (72 h), which was significantly higher than that in the CON group (24.08 ± 2.29 mM) (*p* < 0.0001). The results indicated that the addition of BG136 enhanced the ability of the intestinal microbiota to produce SCFAs. There was no doubt that elevated levels of SCFAs created a more favorable intestinal environment. This is supported by the work of You et al., who demonstrated that short-chain fatty acids were ligands in activating cellular signaling cascades that regulate the gut environment in obesity [42].

The levels of SCFAs in different time duration were detailed in Figure 4B, with only acetic acid and propionic acid revealing statistically significant variations. A particularly noteworthy finding was that the concentration of acetic acid in the BG136 group (7.97 ± 3.74 mM) was significantly lower compared to the CON group (14.49 ± 2.08 mM) at 72 h (Figure 4C). We hypothesize that this result may stem from the interaction between the abundance of specific bacterial genera and the SCFAs content. Notably, at 0 h, short-chain fatty acids were detected in both groups of samples, and propionic acid accounted for the largest proportion of them, indicating that the intestinal flora of the rats used in this experiment mainly produced propionic acid. It can be seen in Figure 4D that the concentration of propionic acid of BG136 group (22.01 ± 1.65 mM) was significantly higher than that of the CON group (5.21 ± 0.54 mM) at 72 h (*p* < 0.0001). Propionic acid primarily exerts energetic effects on normal cells, with a significant proportion being used for gluconeogenesis and inhibiting cholesterol synthesis [43,44].

### 2.5. BG136 Fermentation Reshaped the Composition of Rat Gut Microbiota

The gut microbiota degrades and utilizes polysaccharides while simultaneously undergoing community restructuring mediated by the metabolite of this catabolic process. In recent years, big progress has been made in the field of interactions between polysaccharides and gut microbiota [40]. To explore the effects of BG136 on the gut community composition, we performed 16S rRNA sequencing following in vitro fermentation. This analysis contributes to a deeper understanding of the intricate bacterial communities, including their diversity, structure, and composition [45].

**Figure 5:**
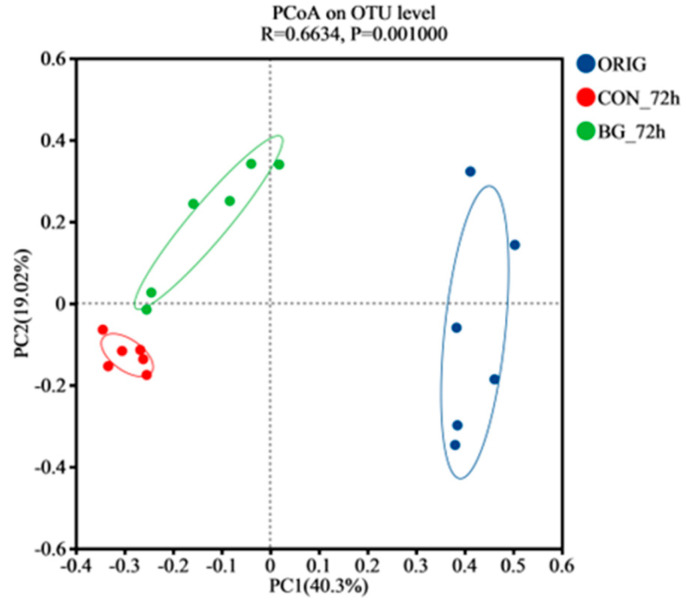
The principal co-ordinates analysis (PCoA) of gut microbiota of the samples. ORIG: the initial gut microbiota without fermentation; CON_72h: fermentation without carbohydrate addition for 72 h; BG_72h: fermentation with BG136 supplement for 72 h.

The rarefaction curves and the Shannon index are widely used to compare species abundance and diversity across samples with varying sequencing depths, while also evaluating the sufficiency of sequencing data [46,47]. As presented in Figure S1, the rarefaction curve flattened with sequencing depth and sample size, confirming sufficient sampling, whereas the Shannon index (Figure S2) revealed higher microbial diversity in the ORIG group. Principal coordinates analysis (PCoA) is a common method used to assess β-diversity, in which the horizontal coordinate (PC1) indicates the direction of the main variation of the sample in the data, while the vertical coordinate (PC2) captures the deviation of the sample in another orthogonal direction. The total variation explained by PC1 (40.3%) and PC2 (19.02%) was 59.32% (Figure 5). Additionally, the results revealed a significant distinction between the microbiota of the original fecal samples (ORIG) and those after 72 h of fermentation, demonstrating substantial microbial community restructuring during the 72 h fermentation process. Clear separations were also observed between CON_72 h and BG_72 h, suggesting that the addition of BG136 influenced the community composition of microbiota.

**Figure 6:**
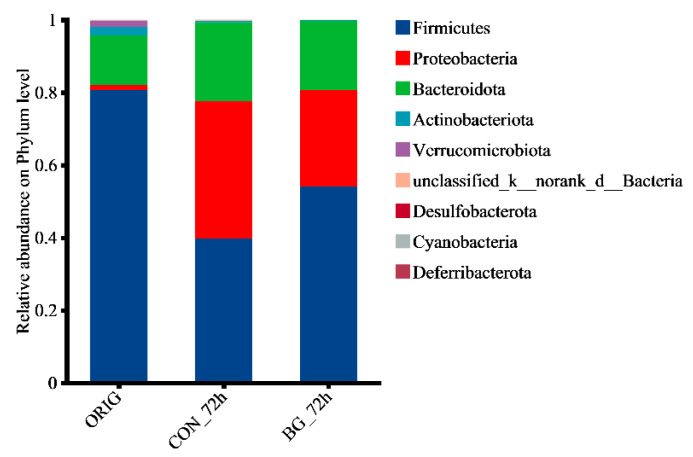
Community barplot analysis in rat fecal samples at the phylum level. ORIG: the initial gut microbiota without fermentation; CON_72h: fermentation without carbohydrate addition for 72 h; BG_72h: fermentation with BG136 supplement for 72 h.

Furthermore, community composition across groups was comparatively analyzed at both phylum and genus levels to visualize specific changes within the gut microbiota. As shown in Figure 6, in the original fecal samples, the phylum with the highest proportion was *Firmicutes*, followed by *Bacteroidota*. Additionally, the samples fermented for 72 h (CON_72 h and BG_72 h) exhibited a higher proportion of *Proteobacteria* and a lower proportion of *Firmicutes*. This could be attributed to the fact that the in vitro fermentation process is not a strictly anaerobic environment, and *Proteobacteria* are facultative anaerobes, while *Firmicutes* are strictly anaerobic [48].

**Figure 7:**
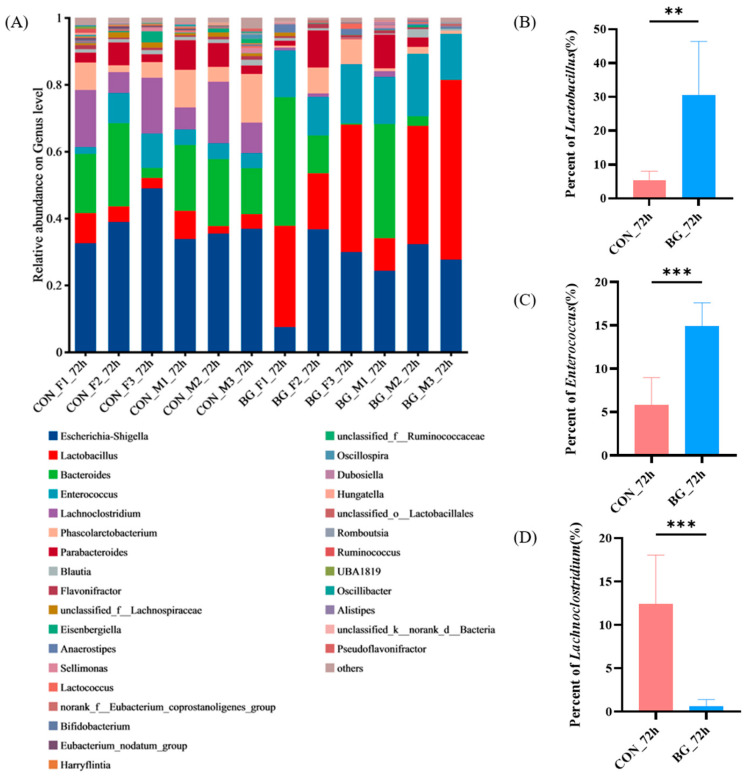
Alterations in intestinal flora due to BG136. (**A**) Comparison of community composition between CON_72 h and BG_72 h at the genus level (*n* = 6). Comparison of the percentage of (**B**) *Lactobacillus*, (**C**) *Enterococcus*, and (**D**) *Lachnoclostridium*. CON_72h: fermentation without carbohydrate addition for 72 h; BG_72h: fermentation with BG136 supplement for 72 h. F: female; M: male. **: *p* < 0.01, ***: *p* < 0.001.

At the genus level, the comparative analysis of community composition between CON_72 h and BG_72 h was shown in Figure 7A. It can be seen from Figure 7B that the BG136 group significantly promoted the proliferation of beneficial bacteria, such as *Lactobacillus* (*p* < 0.01), which plays a key role in maintaining microbial balance, improving digestion, and enhancing immunity [49]. The result was consistent with the previous study reported by Wang et al. [50]. Recent studies have shown that *Lactobacillus* prefers to degrade polysaccharides with low molecular weight and degree of polymerization, subsequently promoting its own growth [51]. Given that BG136 is characterized by its inherently low molecular weight and polymerization degree, the observed significant increase in *Lactobacillus* suggests its potential dominance in BG136 catabolism, leading to subsequent population expansion. However, this proposed mechanism requires further experimental validation. As illustrated in Figure 7C, the BG136 group showed a marked increase in the abundance of *Enterococcus* (*p* < 0.001), which was proved to be capable of immune activation, inhibition of pathogenic bacterial growth, and regulation of intestinal health [52]. For example, Miyazaki et al. found that *Enterococcus* exerted a strong bactericidal effect on *enteroaggregative E. coli* by inducing membrane damage and cytolysis [53]. It was significant to observe that BG136 reduced the proportion of *Lachnoclostridium* in Figure 7D (*p* < 0.001), a genus that has been shown to be inversely correlated with SCFA levels. Ma et al. demonstrated that the abundance of *Lachnoclostridium* was positively correlated with acetic acid levels but negatively correlated with propionic acid levels [54]. However, an increased presence of *Lachnoclostridium* has been associated with the development of colorectal cancer [55]. To further characterize the differences in the composition of intestinal flora among groups, heatmap analysis is presented in Figure S3.

**Figure 8:**
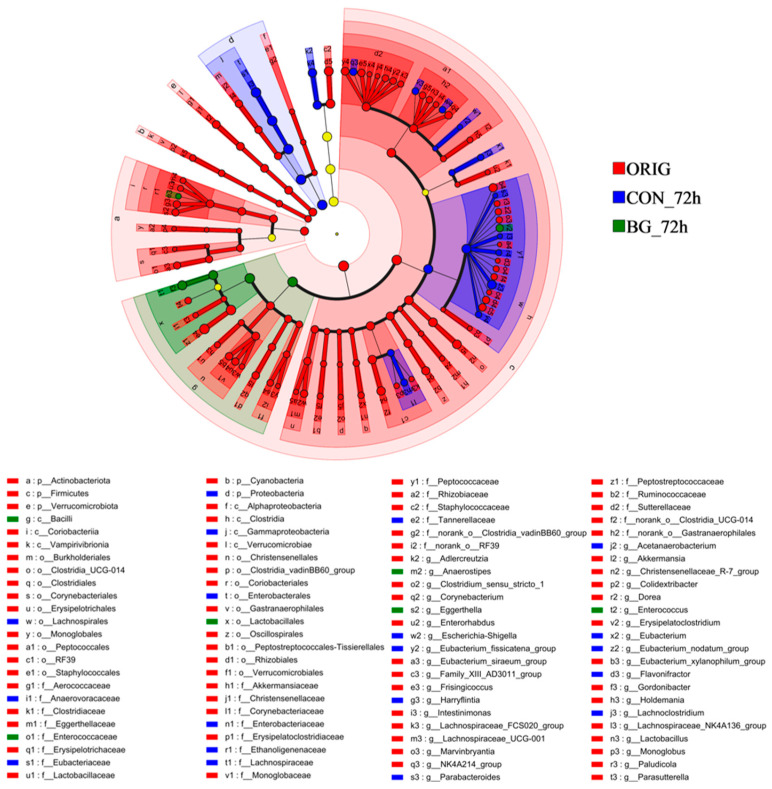
Lefse analysis of gut microbiota. ORIG: the initial gut microbiota without fermentation; CON_72h: fermentation without carbohydrate addition for 72 h; BG_72h: fermentation with BG136 supplement for 72 h.

LEfSe analysis was employed to describe microbiota with significant differences after fermentation. Additionally, linear discriminant analysis (LDA) was used to assess the impact of species on differential effects, with higher LDA scores indicating greater species abundance influence. As highlighted in Figure 8, the BG_72h group exhibited a prominent signature microbiota, including *Bacilli*, *Lactobacillales*, *Enterococcaceae*, and *Enterococcus*. In addition, LDA scores were depicted in Figure S4. Notably, substantial interindividual variations were observed in the gut microbiota composition among the six subjects, which likely accounted for the differences in the degree and rate of fecal fermentation among the individuals (Figure S5). The current investigation demonstrates that BG136 supplementation exerts beneficial modulatory effects on gut microbiota composition. These findings align with evidence highlighting the prebiotic potential of marine-derived polysaccharides in microbial regulation, including laminarin [56,57], fucoidan [58,59,60], and carrageenan [61,62].

## 3. Materials and Methods

### 3.1. Materials and Chemicals

BG136 was provided by Marine Biomedical Research Institute of Qingdao (Qingdao, China). Simulated gastric fluid (USP) and simulated intestinal fluid were acquired from Beijing Leagene Biotech Co., Ltd. (Beijing, China). All other reagents and chemicals used were of analytical grade.

### 3.2. In Vitro Simulated Digestion of BG136

#### 3.2.1. Design for In Vitro Simulated Gastric Fluid Digestion

BG136 solutions at a concentration of 5 mg/mL were prepared, and four equal-volume groups were established. Simulated gastric fluid was added at a ratio of 1:1 (*v*/*v*), and the mixtures were incubated at 37 °C on a constant-temperature shaker for 0, 2, 4, and 6 h. At the end of each incubation period, the enzyme activity was inactivated by boiling the samples for 10 min. The pH was then adjusted to 7.0 using 1 M NaHCO_3_. The blank group (BLANK), consisting of double-distilled water, was incubated for 6 h under the same conditions. Samples (50 μL) were collected at different time points.

#### 3.2.2. Design for In Vitro Simulated Intestinal Fluid Digestion

The sample digested with simulated gastric fluid for 6 h was divided into four equal-volume groups. Simulated intestinal fluid was added at a ratio of gastric digestive solution to simulated intestinal solution of 3:1 (*v*/*v*), and the mixtures were incubated at 37 °C on a constant-temperature shaker for 0, 2, 4, and 6 h. The blank group (BLANK) was a sample of double-distilled water digested with simulated gastric fluid for 6 h. At the end of each incubation period, the enzyme activity was inactivated by boiling the samples for 10 min. A 50 μL aliquot from each sample was then collected for measurement.

#### 3.2.3. HPSEC-MS Detection of BG136 After Digestion

The digested samples were processed as follows: 50 μL of each sample was mixed with equal volumes of 500 mM ammonium acetate solution and 150 μL of methanol. The mixture was vortexed for 30 s and then centrifuged at 14,000 rpm for 10 min. The supernatant was subsequently ultrafiltered multiple times through a 3 kDa cutoff ultrafiltration column, and the filtrate was collected for analysis using a Thermo LTQ Orbitrap XL mass spectrometer (Thermo Fisher Scientific, Waltham, MA, USA). A TSKgel UP-SW3000 column (2 μm, 4.6 mm I.D. × 30 cm, Tosoh Bioscience, Tokyo, Japan) was used as the analytical column, with a column temperature set at 40 °C. The mobile phase consisted of 5 mM ammonium acetate in methanol (90:10, *v*/*v*), and the samples were eluted at a flow rate of 0.15 mL/min for 35 min. The ion source for mass spectrometry was ESI; negative ion mode; resolution: 30,000; mass range: 200–3000 Da. Mass spectrometry analysis was performed using Xcalibur 4.2 software.

### 3.3. In Vitro Simulated Fermentation of BG136

#### 3.3.1. Preparation of Fecal Fermentation Media

The basal medium was prepared as follows: 3.0 g of tryptone, 3.0 g of peptone, 4.5 g of yeast extract, 4.5 g of NaCl, 2.5 g of KCl, 4.5 g of MgCl_2_·6H_2_O, 0.2 g of CaCl_2_·6H_2_O, 0.4 g of KH_2_PO_4_, 1 mL of Tween-80, 0.5 g of mucin, 10 mL of hemoglobin, 200 μL of trace elements, and 0.8 g of L-cysteine hydrochloride were dissolved in 1 L of double-distilled water. The experiment consisted of two groups: the control group (CON) and the experimental group (BG136). To the experimental group, 5 mg/mL of BG136 was added, while no carbon source was added to the control group. After preparing the medium, the pH was adjusted to 6.4–6.5 with NaOH, and the medium was then purged with nitrogen and sterilized at 121 °C.

#### 3.3.2. Preparation of Fecal Suspension and Fermentation

Fresh feces from 6-week-old healthy Sprague-Dawley (SD) rats (3 males and 3 females) were collected, with a portion immediately frozen as an unfermented control (ORIG) for subsequent testing. The fresh fecal samples were then mixed with sterilized double-distilled water to prepare six 10% (*w*/*v*) fecal suspensions. These suspensions were left to settle naturally, allowing insoluble particles to separate. The supernatant was then added to the medium of both the control and experimental groups at a 1:10 (*v*/*v*) ratio and incubated at 37 °C. At 0, 6, 12, 24, 48, and 72 h, a certain volume of the fermentation broth was collected for subsequent analysis. The entire experimental procedure was conducted in an anaerobic workstation (AW500, Electrotek, West Halifax, UK).

#### 3.3.3. TLC Analysis of BG136 Degradation

To determine whether the intestinal flora of rats can degrade and utilize BG136, TLC was performed on a silica plate (Macherey-Nagel, Düren, Germany) using a solvent mixture of n-butanol, formic acid, and water (4:6:1, *v*/*v*). Fermentation samples were centrifuged at 14,000 rpm for 10 min, and the supernatant was applied to the silica plate. Each sample was spotted twice, with 0.5 μL applied each time. The spots were visualized by spraying with aniline-diphenylamine-phosphoric acid solution and then baked at 110 °C.

#### 3.3.4. Determination of Total Sugar, Reducing Sugar, and Calculation of Fermentability

Total sugar content was assessed using the phenol-sulfuric acid assay, while the content of reducing sugars (CR) was determined using the 3,5-dinitrosalicylic acid (DNS) method. The fermentability (%) of BG136 was calculated using the following method:

(1) $$\mathrm{Fermentability}\;\left(\%\right)=\frac{\;1-\left(\mathrm{Total}\;\mathrm{sugar}\;\mathrm{after}\;\mathrm{fermentation}-\mathrm{Reducing}\;\mathrm{sugar}\right)}{\mathrm{Total}\;\mathrm{sugar}\;\mathrm{after}\;\mathrm{fermentation}}\;\times \;100\%$$

#### 3.3.5. HPSEC-MS Detection of BG136 After Fermentation

The samples were the fermentation broths described in Section 3.3.2, and the sample handling and detection methods were the same as those used in Section 3.2.3.

#### 3.3.6. Determination of Short-Chain Fatty Acids

A series of standard solutions of SCFAs at working concentrations were prepared, including acetic acid, propionic acid, butyric acid, valeric acid, isovaleric acid, lactic acid, and isobutyric acid. The samples were prepared by mixing 100 μL of fermentation broth with 100 μL of 1% H_2_SO_4_. The mixture was shaken vigorously, and the supernatant was obtained by centrifuging at 14,000 rpm for 10 min, followed by filtration through a 0.22 μm membrane filter for detection.

The concentrations of SCFAs were determined using an Agilent 1260 HPLC system (Agilent, Santa Clara, CA, USA) equipped with an Aminex HPX-87H Ion Exclusion Column (9 μm, 300 mm × 7.8 mm, Bio-Rad Laboratories, Hercules, CA, USA). The column temperature was set to 50 °C. The samples were eluted with a mobile phase of 5 mM H_2_SO_4_ at a flow rate of 0.6 mL/min for 40 min. The detection wavelength was set to 210 nm, with a detection bandwidth of 3 nm.

#### 3.3.7. Analysis of Gut Microbiota

After 72 h of fermentation, the fermentation broths (CON_72 h and BG_72 h) along with unfermented samples (ORIG) were collected and sent to Shanghai Majorbio Bio-Pharm Technology Co., Ltd. (Shanghai, China) for high-throughput sequencing and bioinformatics analysis. The sequencing was conducted using the Illumina MiSeq PE300 platform (San Diego, CA, USA). Subsequent analysis was performed using the Majorbio Cloud Platform (https://www.majorbio.com/).

### 3.4. Statistical Analysis

All experimental data are presented as mean ± standard deviation. ANOVA, followed by Duncan’s test for multiple comparisons, was used for data analysis. A *p*-value of ≤0.05 was considered statistically significant.

## 4. Conclusions

To preliminarily characterize the gastrointestinal metabolism of orally administered BG136, in vitro digestion and fermentation models were employed to simulate its biotransformation processes, revealing that BG136 was transported intact to the colon where it underwent microbial-mediated degradation. Additionally, substantial individual variability was observed in the fermentation degree of BG136, potentially due to differences in the initial community structures between individuals. In addition, we explored the beneficial effects of BG136 on the intestinal tract, including the generation of SCFAs and the regulation of intestinal flora. The results showed that intestinal flora stimulated by BG136 were able to produce more abundant SCFAs, accompanied by a significant increase in propionic acid and a decrease in acetic acid. In addition, BG136 restructured the bacterial flora composition, as evidenced by a significant increase in the proportions of *Lactobacillus* and *Enterococcus*, and a marked decrease in *Lachnoclostridium*. In summary, the current findings demonstrate that BG136 is indigestible but undergoes biotransformation by gut flora, exhibiting considerable potential for promoting intestinal health. This study has a direct translational implication for developing the oral pharmaceutical formulation of BG136 and providing a scientific basis for positioning BG136 as a novel prebiotic supplement. Specifically, the drug’s inherent stability against upper gastrointestinal degradation offers substantial manufacturing advantages, particularly by obviating the requirement for specialized formulation strategies like enteric coating systems, thereby streamlining production workflows and significantly reducing associated costs. Additionally, the observed interindividual variations in microbial bioconversion efficiency highlight the need for personalized dosage regimens guided by microbiome profiling.

## Data Availability

Data will be made available upon request.

## Supplementary Materials

The following supporting information can be downloaded at: https://www.mdpi.com/article/10.3390/md23040156/s1, Figure S1: The rarefaction curves of gut microbiota of the samples; Figure S2: The Shannon indexes of gut microbiota of the samples; Figure S3: Heatmap analysis of the gut microbiota at the genus level; Figure S4: LDA analysis of gut microbiota; Figure S5: Initial gut microbiota composition of different individuals at the genus level.
