# Supplementary material for: In Vitro Digestion and Gut Microbiota Fermentation of the Anticancer Marine Drug BG136: Stability and Biotransformation Investigation
Source: Mar Drugs. 2025 Apr 3;23(4):156. doi: 10.3390/md23040156 (PMC12028602; doi:10.3390/md23040156)
Supplement: Supplementary file 1 [file marinedrugs-23-00156-s001.zip › marinedrugs-3539436-supplementary.pdf]

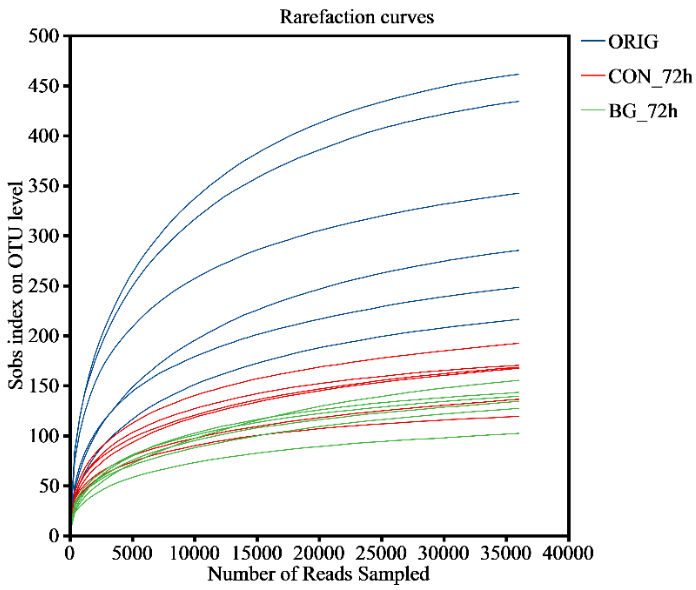

Figure S1. The rarefaction curves of gut microbiota of the samples. ORIG: the initial gut microbiota without fermentation; CON_72h: fermentation without carbohydrate addition for 72 h; BG_72h: fermentation with BG136 supplement for 72 h.

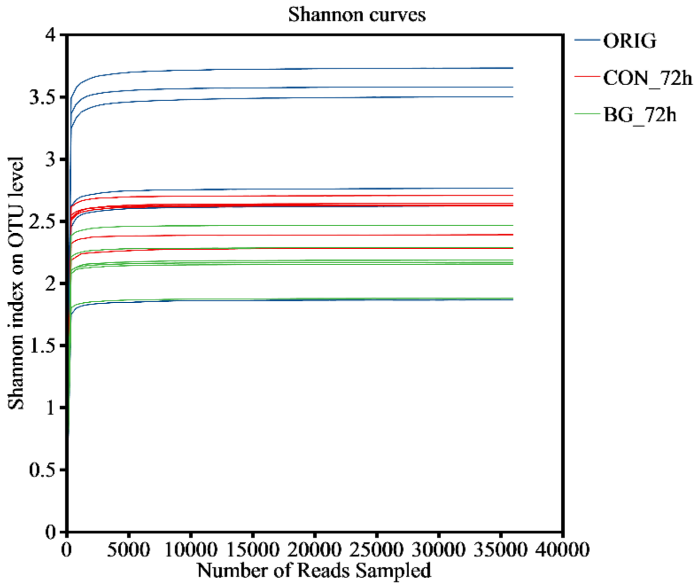

Figure S2. The Shannon indexes of gut microbiota of the samples. ORIG: the initial gut microbiota without fermentation; CON_72h: fermentation without carbohydrate addition for 72 h; BG_72h: fermentation with BG136 supplement for 72 h.

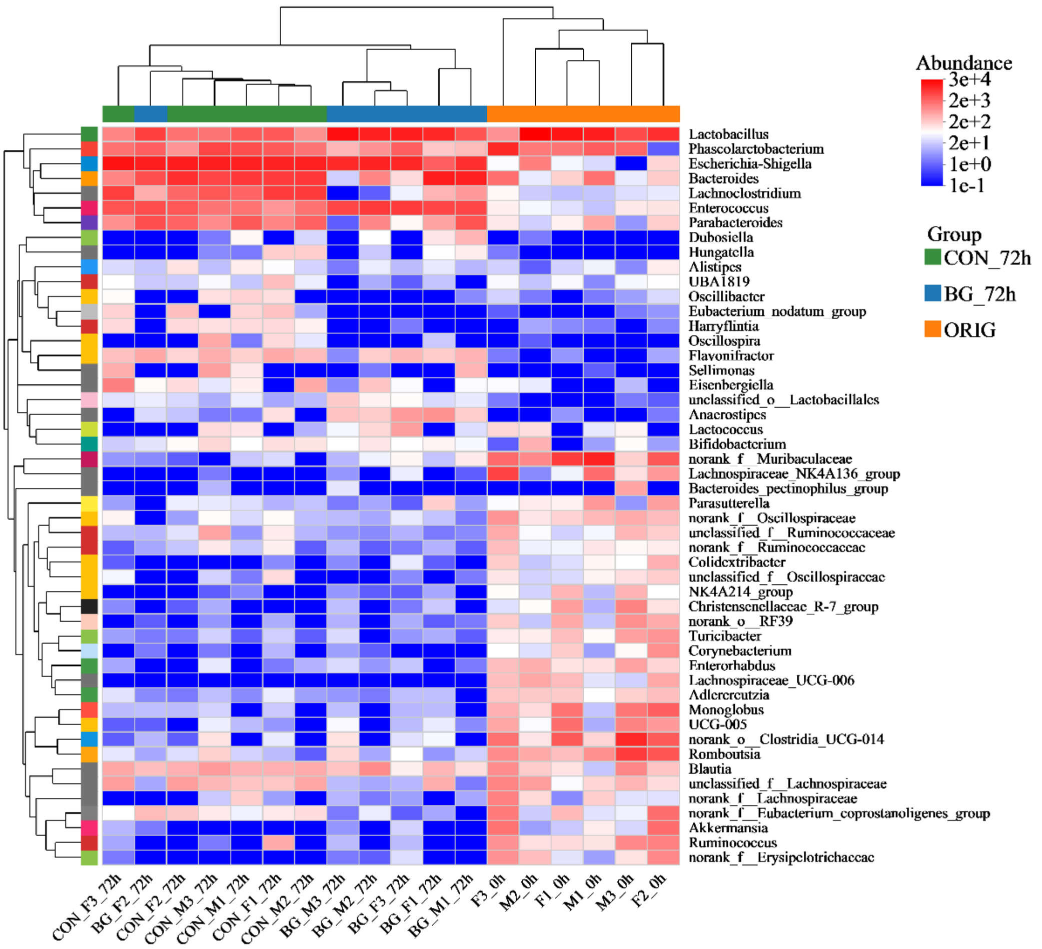

Figure S3. Heatmap analysis of gut microbiota at the genus level . ORIG: the initial gut microbiota without fermentation ; CON_72h: fermentation without carbohydrate addition for 72 h; BG_72h: fermentation with BG136 supplement for 72 h. F: female; M:male.

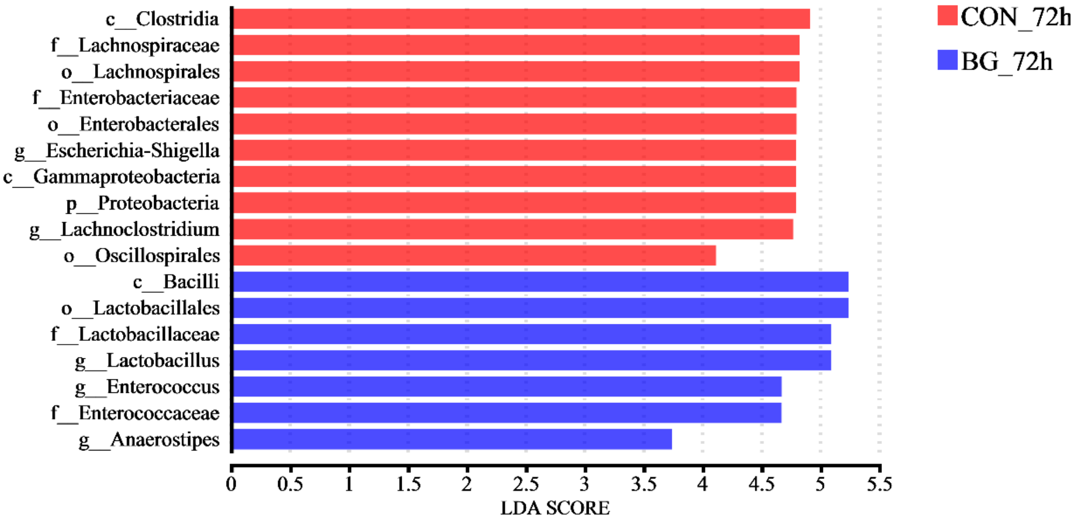

Figure S4. LDA analysis of gut microbiota. CON_72h: fermentation without carbohydrate addition for 72 h; BG_72h: fermentation with BG136 supplement for 72 h.

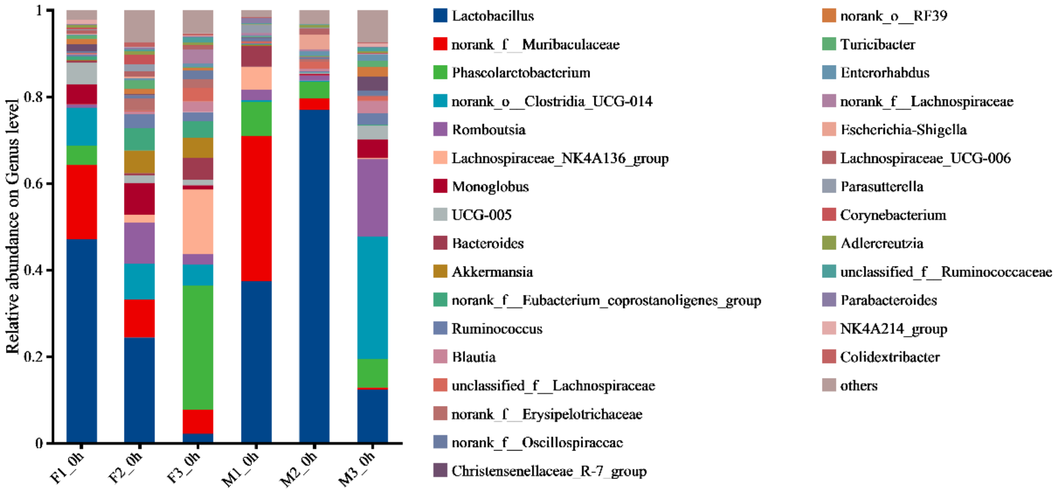

Figure S5. Initial gut microbiota composition of different individuals at the genus level. F: female; M: male.
